# Interactions of the CpxA sensor kinase and cognate CpxR response regulator from *Yersinia pseudotuberculosis*

**DOI:** 10.1186/1756-0500-5-536

**Published:** 2012-09-27

**Authors:** Edvin J Thanikkal, Jagadish C K Mangu, Matthew S Francis

**Affiliations:** 1 Department of Molecular Biology, Umeå University, Umeå, SE-901 87, Sweden; 2 Umeå Centre for Microbial Research, Umeå University, Umeå, SE-901 87, Sweden

**Keywords:** BACTH assay, λCI homodimerization assay, Homodimer, Heterodimer, Linker, Coiled-coil, Winged helix-turn-helix, Phosphorylation

## Abstract

**Background:**

The CpxA sensor kinase-CpxR response regulator two-component regulatory system is a sentinel of bacterial envelope integrity. Integrating diverse signals, it can alter the expression of a wide array of components that serve to shield the envelope from damage and to promote bacterial survival. In bacterial pathogens such as *Yersinia pseudotuberculosis*, this also extends to pathogenesis. CpxR is thought to dimerize upon phosphorylation by the sensor kinase CpxA. This phosphorylation enables CpxR binding to specific DNA sequences where it acts on gene transcription. As Cpx pathway activation is dependent on protein-protein interactions, we performed an interaction analysis of CpxR and CpxA from *Y. pseudotuberculosis*.

**Results:**

CpxR full-length and truncated versions that either contained or lacked a putative internal linker were all assessed for their ability to homodimerize and interact with CpxA. Using an adenylate cyclase-based bacterial two hybrid approach, full-length CpxR readily engaged with CpxA. The CpxR N-terminus could also homodimerize with itself and with a full-length CpxR. A second homodimerization assay based upon the λcI repressor also demonstrated that the CpxR C-terminus could homodimerize. While the linker was not specifically required, it enhanced CpxR homodimerization. Mutagenesis of *cpxR* identified the aspartate at residue 51, putative N-terminal coiled-coil and C-terminal winged-helix-turn-helix domains as mediators of CpxR homodimerization. Scrutiny of CpxA full-length and truncated versions revealed that dimerization involved the N-terminus and an internal dimerization and histidine phosphotransfer domain.

**Conclusions:**

This interaction analysis mapped regions of CpxR and CpxA that were responsible for interactions with self or with each other. When combined with other physiological and biochemical tests both hybrid-based assays can be useful in dissecting molecular contacts that may underpin Cpx pathway activation and repression.

## Background

Conditions that threaten the integrity of the bacterial envelope are collectively termed extracytoplasmic stresses (ECS). Bacteria employ a series of ECS-responsive regulatory pathways to control the expression of ‘survival’ genes whose products act in the periplasm to maintain membrane integrity. This ensures continued bacterial growth even in environments poisoned by harmful ECS. A notable ECS-responsive pathway is the CpxA-CpxR two-component regulatory system (TCRS) [1,2]. Belonging to the class I histidine kinases [3], CpxA is the integral inner-membrane sensor kinase (SK). Upon activation by ECS sensing – presumably in the form of misfolded proteins – it becomes auto-phosphorylated. Through a phospho-transfer reaction, CpxA transduces this signal through the membrane to activate the cytoplasmic CpxR response regulator (RR). CpxR belongs to the OmpR/PhoB family of winged-helix-turn-helix (wHTH) transcriptional RRs [4]. Phosphorylated CpxR (CpxR ~ P) then binds to the promoters of genes coding for several protein folding and degradation factors that operate in the periplasm. An important function of the Cpx pathway is therefore in protein quality control in the bacterial envelope with an emphasis on maintaining outer membrane structural integrity [1,2]. However, as the CpxR regulon may include hundreds of genes [5,6], playing sentinel to the cell envelope must incorporate diverse signals and safeguarding mechanisms.

In Gram negative enteropathogenic *Yersinia pseudotuberculosis,* activation of the Cpx pathway also regulates periplasmic protein folding and degradation factors [7-10]. Additionally, accumulation of CpxR ~ P down-regulates several prominent *Yersinia* virulence determinants. Most notable is the Ysc-Yop type III secretion system [7,10], the cellular adhesin known as invasin and its transcriptional activator RovA [8,9]. RovA is a global regulator in pathogenic *Yersinia* and is capable of influencing the expression of at least 60 genes [11-13]. Hence, the regulatory influence of CpxR ~ P – either direct or indirect via effects on *rovA* expression – has potential to be quite widespread in these bacteria.

An activated Cpx pathway may therefore function to restrict *Y. pseudotuberculosis* virulence factor expression during times of ECS when all resources must be dedicated to the expression of survival genes. In the absence of ECS, virulence factor expression can be de-repressed to permit *Y. pseudotuberculosis* to mount a successful host infection [14]. In fact, accumulating evidence in a few Gram-negative pathogens suggests that the Cpx pathway might possess a universal role in virulence factor regulation and bacterial pathogen fitness either by aiding in the establishment of an environmental reservoir or during host infections [2,15-17].

**Figure 1:**
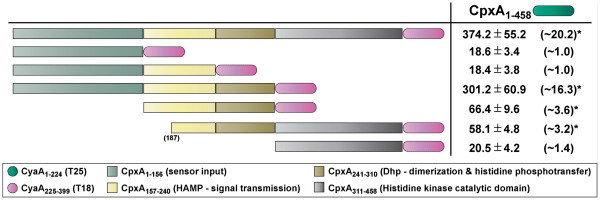
**BACTH analysis of CpxA**-**CpxA interactions.** Full-length CpxA_1-458_ was translationally fused to the N-terminus of CyaA_1-224_ (T25 – dark green shade) creating a CpxA_1-458_-T25 hybrid used as the ‘bait’. Full-length CpxA_1-458_ was also translationally fused to the N-terminus of CyaA_225-399_ (T18 – magenta shade) giving rise to a ‘prey’ CpxA_1-458_-T18 hybrid. Based on CpxA divisions into sensor input (CpxA_1-156_, cadet blue shade), HAMP – signal transmission (CpxA_157-240_, soft yellow shade), Dhp – dimerization and histidine phosphorylation (CpxA_241-310_, metallic gold shade) and histidine kinase catalysis (CpxA_311-458_, grey shade) domains, an additional six ‘prey’ CpxA-T18 hybrids were constructed; CpxA_1-156_-T18, CpxA_1-240_-T18, CpxA_1-310_-T18, CpxA_157-310_-T18, CpxA_187-458_-T18 and CpxA_311-458_-T18. BACTH interaction analysis of ‘bait’ and ‘prey’ hybrids was quantified via measurement of β-galactosidase activity and is represented as units/mg dry weight of host *E. coli* BTH101 bacteria (left column; black font). As an internal positive control, we used the provided constructs expressing T18-Zip and T25-Zip that yielded 1547.0 ± 121.2 units of β-galactosidase activity /mg dry weight of bacteria. This was equivalent to ~83.8 fold more enzymatic activity produced compared to bacteria co-expressing only T18 and T25 (18.5 ± 3.9 units of β-galactosidase activity). The fold change in enzymatic activity caused by CpxA-CpxA interactions relative to this negative control is indicated in parentheses to the right. A level of β-galactosidase activity at least 3-fold higher than the negative control was considered to indicate a positive interaction (*). Data is presented as the mean (± standard error of the mean) of at least four independent experiments performed in triplicate.

CpxA possesses a modular structure [3,18,19] (Figure 1). Consecutive domains in their N-terminus are responsible for signal input, processing and signal transmission. The latter is defined as the HAMP linker domain by virtue of its presence in Histidine kinases, Adenyl cyclases, Methyl-accepting proteins and Phosphatases [20]. Located in the cytoplasm, the HAMP linker domain most likely regulates the phosphorylation of histidine SKs by transmitting conformational changes originating in the periplasmic ligand-binding domains to the cytoplasmic-located C-terminal kinase catalytic domain [20,21]. The remainder of the protein is composed of an internal dimerization and histidine phosphotransfer (DHp) domain and then a C-terminal histidine kinase catalytic domain [22,23]. Together, these two consecutive domains form the kinase core. The DHp domain mediates dimerization [24], and possesses the conserved phospho-accepting histidine residue and also a phosphatase domain for dephosphorylating CpxR [22,23,25,26]. Lastly, the catalytic domain is essential for kinase activity. It contains a number of conserved motifs necessary for ATP binding [27,28] and probably also for catalysis and phosphotransfer [22]. 

**Figure 2:**
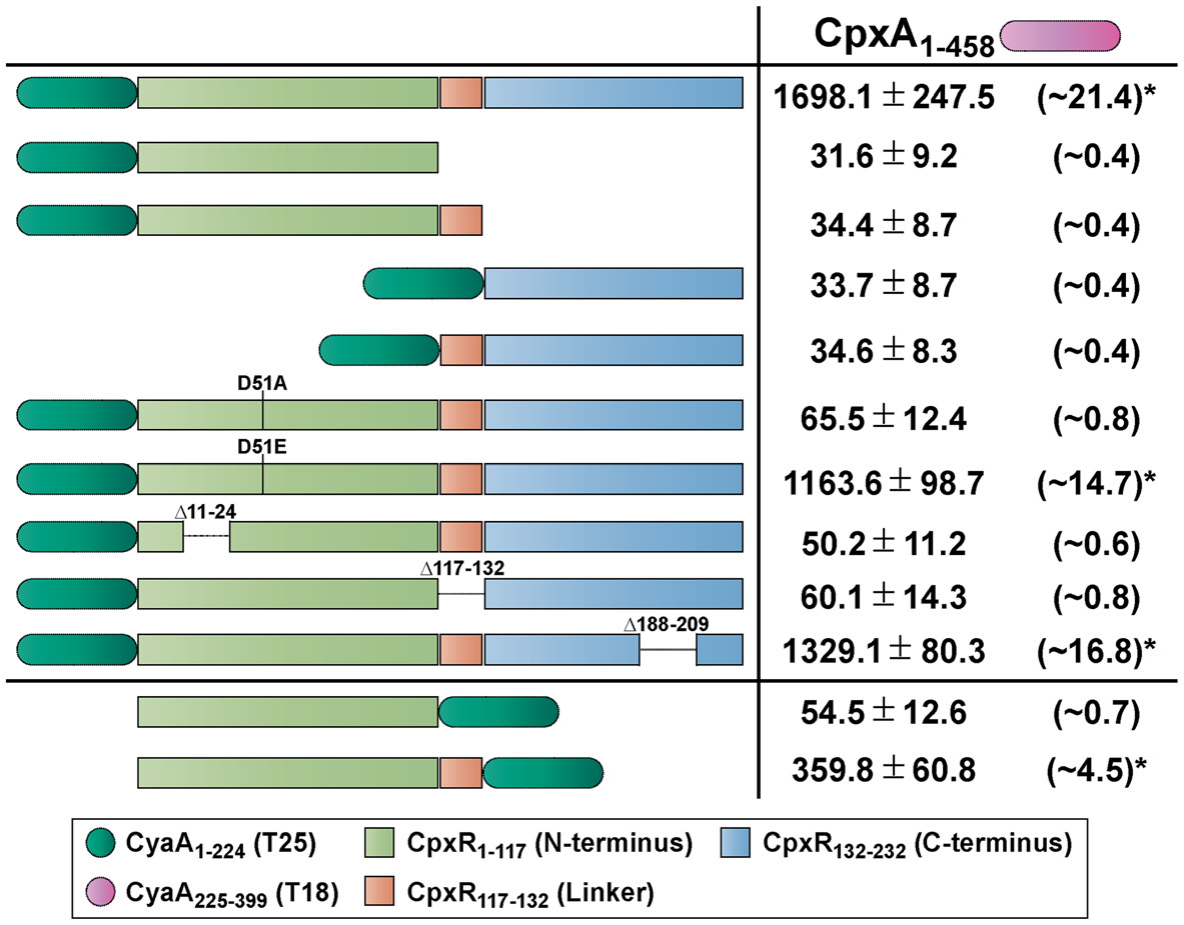
**Regions of CpxR interacting with CpxA as monitored by BACTH analysis.** Full-length CpxA_1-458_ was translationally fused to the N-terminus of CyaA_225-399_ (T18 – magenta shade) giving rise to a ‘bait’ CpxA_1-458_-T18 hybrid. Full-length CpxR_1-232_ was translationally fused to the C-terminus of CyaA_1-224_ (T25 – dark green shade) giving rise to a T25-CpxR_1-232_ ‘prey’ hybrid. CpxR was also divided into N-terminal (pistachio green), internal linker (orange) and C-terminal (sky blue) domains. Based on these divisions, additional ‘prey’ T25 hybrids were similarly constructed that consisted of only the N-terminus without linker (T25-CpxR_1-117_) or with linker (T25-CpxR_1-132_) and the C-terminus without linker (T25-CpxR_132-232_) or with linker (T25-CpxR_117-232_). CpxR_1-132_ was also fused to the N-terminus of T25 giving the CpxR_1-132_-T25 hybrid. Full-length or near full-length CpxR ‘prey’ T25 constructs were also generated consisting of T25-CpxR_D51A_ or T25-CpxR_D51E_ lacking the phosphorylated aspartate residue as well as T25-CpxR_Δ11–24_, T25-CpxR_Δ117–132_ and T25-CpxR_Δ188–209_ lacking the putative N-terminal coiled-coil, internal linker and C-terminal winged helix-turn-helix regions respectively. BACTH interaction analysis of ‘bait’ and ‘prey’ hybrids was quantified via measurement of β-galactosidase activity and is represented as units/mg dry weight of host *E. coli* BTH101 bacteria (left column; black font). The internal positive control based upon constructs expressing T18-Zip and T25-Zip yielded 1603.6 ± 146.4 units of β-galactosidase activity/mg dry weight of bacteria. This was equivalent to ~20.2 fold more enzymatic activity produced compared to bacteria co-expressing only T18 and T25 (79.2 ± 6.2 units of β-galactosidase activity). The fold change in enzymatic activity caused by CpxA-CpxR interactions relative to this negative control is indicated in parentheses to the right. The asterisks (*) indicates a positive interaction. Data is presented as the mean (± standard error of the mean) of at least four independent experiments performed in triplicate

CpxR contains two structurally conserved domains; a N-terminal receiver domain and a C-terminal effector domain that are joined by a flexible internal linker [29,30] (Figure 2). The N-terminal receiver domains of RRs share several conserved features such as a α_4_-β_5_-α_5_ interface and an enrichment in aspartate/serine/threonine amino acids. These residues are thought to cooperate in dimerization as a result of phosphorylation induced conformational changes that in turn propagates the signal [29,30]. A common wHTH motif defines the C-terminal effector domain. This motif is responsible for binding to target DNA in order to regulate transcription output [29,30]. The internal linker region that tethers together the receiver and effector domain might as well contribute in signal propagation, perhaps by promoting necessary molecular interactions [29,30]. 

Significantly, TCRS are a potential target for the development of novel anti-bacterial therapeutics [31], which are urgently needed to overcome the current public health crisis associated with common antibiotic resistances among prominent bacterial pathogens [32]. Given the emerging theme of CpxA-CpxR-dependent virulence gene regulation in clinically important bacteria [2,15-17], this pathway might well be an attractive target for chemical modulation. With this in mind, the present study is primarily aimed at establishing practical and reliable assays to identify the molecular interactions of CpxA and CpxR from pathogenic *Y. pseudotuberculosis*. Not only would this guide further *in vivo* mutagenesis studies to explore the molecular determinants involved in CpxA-CpxR signal transduction and virulence gene control in *Y. pseudotuberculosis,* this knowledge should also benefit studies aimed at targeting the Cpx pathway using chemical manipulation.

## Methods

### Bacterial strains, plasmids and growth conditions

Bacterial strains and plasmids are listed in Supplementary Table S1 and available for online download as Additional file 1. Unless indicated, all bacteria were routinely cultured in Luria-Bertani (LB) broth or agar at 37°C for *E. coli* or 26°C for *Y. pseudotuberculosis.* When required, antibiotics at the following final concentrations were used: ampicillin (Ap; 100 μg/ml), kanamycin (Km; 25 μg/ml), chloramphenicol (Cm; 25 μg/ml), tetracycline (Tc; 10 μg/ml).

### Molecular methods

DNA fragments were PCR amplified from YPIII/pIB102 (parental *Y*. *pseudotuberculosis*) using Easy-A High Fidelity PCR cloning enzyme (Agilent technologies, Santa Clara, California, USA) and the oligonucleotides pairs synthesized by Sigma-Aldrich Sweden AB (Stockholm, Sweden) and are listed in Supplementary Table S2 (available for online download as Additional file 2). Amplified DNA fragments were cloned into pTZ57T/R (Fermentas, Vilnius, Lithuania) and correct sequence were verified by Eurofins MWG Operon (Ebersberg, Germany). Confirmed sequences were then re-cloned as a translational fusion into the XbaI/EcoRI site of the pKT25, pKNT25, pUT18, or pUT18C vectors for BACTH analysis or the BglII/KpnI site of the pKWY2428 vector for λcl homodimerization analysis. Routine maintenance of individual clones was performed in *E. coli* DH5.

### BACTH and the β-galactosidase assay

Pairs of BACTH vectors expressing CyaA T18 and T25 fusions to ‘bait’ and ‘prey’ proteins were sequentially transformed into *E. coli* BTH101 using the chemical transformation method [33]. Transformants were selected at 37°C by overnight growth on LB agar with appropriate antibiotics and supplemented with 0.5 mM isopropyl-β-d-thiogalactopyranoside (IPTG) and 40 μg/ml 5-bromo-4-chloro-3-indolyl-β-d-galactopyranoside (X-Gal). Well isolated single colonies were then selected for inoculation of bacterial cultures that were grown overnight at 30°C in LB broth supplemented appropriate antibiotics. Then 0.025 volumes were sub-cultured in the same fresh media with 0.5 mM IPTG and grown for a further 2 h at 30°C. The remainder of the β-galactosidase assay followed descriptions outlined in the BACTH manual and associated literature (Euromedex, Souffelweyersheim, France) [34]. β-galactosidase activity was represented in Units/mg dry weight bacteria according to the manufacturer’s direction. Data are a representative of at least four independent experiments performed in triplicate. Guided by previous analyses [35], we considered a positive interaction only if the β-galactosidase activity level was at least three-fold higher than that measured for the negative control plasmids (expressing T18 and T25 alone).

For western blotting, similarly grown cultures were harvested after late-logarythmic growth. Pellets were solubilized in loading buffer (50 mM Tris–HCl pH 6.8, 2% SDS, 10% Glycerol, 1% β-Mercaptoethanol and 0.02% Bromophenol blue) and fractionated by SDS-PAGE with 12% acrylamide. Protein was then transferred to Immobilon®-P PDVF transfer membrane (Millipore Corporation) using a Hoefer semi-dry transfer apparatus (GE Healthcare). Membranes were exposed to rabbit polyclonal antibodies that were a gift from Thomas Silhavy (anti-CpxA), produced by Agrisera AB (Vännäs, Sweden) from purified antigen (anti-CpxR) or purchased from Santa Cruz Biotechnology Inc [(anti-CyaA (b-300)]. These were then detected with an anti-rabbit monoclonal antibody conjugated with horse radish peroxidase (GE Healthcare) and Pierce ECL 2 western blotting substrate (Thermo Scientific).

### λcI Homodimerization assays

As performed previously [36], assays were carried-out in the *E. coli* JM109 background. In brief, freshly transformed bacteria were grown overnight at 37°C in LB broth with chloramphenicol selection in the presence of 0.1 mM IPTG. Next, 0.01 volumes were sub-cultured in fresh media and grown to mid-logarithmic phase of growth. All cultures were standardized according to an optical density of 600 nm (OD_600_) and 5 μl suspensions of 10-fold serial dilutions were spotted onto LB agar with chloramphenicol, tetracycline and 0.1 mM IPTG. The ability to grow in the presence of tetracycline was scored following overnight incubation at 37°C for at least four independent experiments.

Additionally, λcl-CpxR fusions contained within these pelleted bacterial *E. coli* cells were fractionated by SDS-PAGE with 12% acrylamide. Protein was then transferred to Protran® nitrocellulose transfer membrane (Whatman GmbH) using a Hoefer semi-dry transfer assembly (GE Healthcare). Proteins of interest were bound with specific rabbit polyclonal anti-CpxR antibodies and then detected with an anti-rabbit monoclonal antibody conjugated with horse radish peroxidase and a homemade chemiluminescent solution. As a loading control, we utilized specific rabbit polyclonal anti-CAT antibodies (Sigma-Aldrich) to detect levels of chloramphenicol acetyltransferase encoded by the *cat* gene located on the vector pKWY2428.

## Results

### Dimerization of the CpxA sensor kinase using the BACTH assay

This study had the goal to identify a cost-effective, reliable and convenient protein-protein interaction assay for the characterization of regulatory deficient mutants of CpxA and CpxR from *Y. pseudotuberculosis*. The BACTH system – involving reconstitution of *Bordetella pertussis* adenylate cyclase (CyaA) T18 and T25 domains – is reported to be ideal for analyzing the interactions among membrane-anchored bacterial proteins in their natural environment [34,35], including diverse SKs [37-41]. Since CpxA is expected to dimerize [1,42], we first analyzed the ability of CpxA from *Y. pseudotuberculosis* to interact with itself in the BACTH assay. A series of constructs composed of N-and C-terminal T18 and T25 translational fusions with CpxA were established. Pairs of plasmids expressing a ‘bait’ and ‘prey’ hybrid were transformed into *E. coli* BTH101. This strain contains a *lacZ* reporter gene under CAP-cAMP control; an interaction between bait and prey will reconstitute CyaA activity leading to accumulation of cAMP that, together with CAP, will stimulate transcription of the *lacZ* reporter gene that is indirectly measurable via an elevation in β-galactosidase activity. Serving as bait, full-length CpxA translationally fused to the N-terminus of T25 (CpxA_1-458_-T25) could clearly bind to the prey hybrids CpxA_1-458_-T18 and C-terminal truncated CpxA_1-310_-T18, as a result producing β-galactosidase activity that was ~20.2 and ~16.3 fold higher than the negative control (Figure 1). Modest increases in β-galactosidase activity of ~3.6 and ~3.2 fold were also indicative of a slight interaction between bait and the respective prey hybrids CpxA_157-310_-T18 and CpxA_187-458_-T18 (Figure 1). Since the DHp domain (residues 241–310) is a common feature of all four interacting hybrids, these data demonstrate its importance to CpxA homodimerization. Corroborating this, the remaining three hybrids (CpxA_1-156_-T18, CpxA_1-240_-T18 and CpxA_311-458_-T18) all lacking the DHp domain produced low reporter activity indicative of poor homodimerization capacity (Figure 1). Although CpxA homodimerization was easily measurable via BACTH analysis, production of β-galactosidase activity was notably lesser than that achieved by a strain having the T25-Zip/T18-Zip positive control plasmids, where accumulated activity was ~83.8 fold more than background (data not shown).

It is possible that low reporter activity might also be due to unstable fusion product. To investigate this, we made numerous attempts to measure protein levels in each fusion bearing strain using western blotting with either anti-CyaA (b-300) or anti-CpxA antibodies. However, neither antibody was successful in detecting accumulated steady state levels of any specific T25 or T18 fusion, even for those fusion-bearing strains that displayed high levels of reporter activity and the positive control (data not shown). Hence, for those three strains where low reporter activity was recorded, we cannot definitively conclude that this was due to genuinely poor interactions.

### An N-terminal region of CpxR interacts with CpxA

Our next goal was to examine if the BACTH assay could support CpxR-CpxA interaction studies. In this analysis, we used the full-length CpxA_1-458_-T18 hybrid as bait that in Figure 1 resulted in high reporter activity and must therefore be stably produced. A corresponding prey construct that is composed of T25 C-terminally fused to full-length CpxR (T25-CpxR_1-232_) was co-transformed into *E. coli* BTH101. An interaction between bait and prey yielded β-galactosidase activity that was ~21.4 fold higher than the negative control and equivalent to the positive control (~20.2 fold increase) (Figure 2, data not shown).

To identify the region of CpxR engaging with CpxA, we were guided by a recent study by Tapparel and colleagues [43]. It was suggested that the C-terminal domain effector activity of *E. coli* CpxR can become constitutive by removing the N-terminal regulatory domain appended via an internal flexible linker. We therefore established a series of prey constructs with T25 that were C-terminally fused to truncated CpxR derivatives composed of only the N-terminus without internal linker (T25-CpxR_1-117_) or with internal linker (T25-CpxR_1-132_) and the C-terminus without internal linker (T25-CpxR_132-232_) or with internal linker (T25-CpxR_117-232_). No interaction between any of these prey hybrids and the CpxA_1-458_-T18 bait was observed (Figure 2). Attempts to detect any accumulated T25-CpxR variant in cell lysates by western blotting with either anti-CyaA (b-300) or anti-CpxR antibodies were again unsuccessful, even for the full-length CpxR fusion that did interact with CpxA (data not shown). On the other hand, altering the orientation of the T25 fusion by creating an alternative prey hybrid of CpxR_1-132_-T25 did permit modest reporter activity (~4.5 fold) indicative of an interaction with the CpxA_1-458_-T18 bait (Figure 2). Thus, this suggested that the CpxR N-terminus might be more important for CpxA binding.

To explore this further, we utilized a CpxR_D51A_ variant that is non-phosphorylatable and is therefore essentially inactive (‘locked off’) [9]. Additionally, another non-phosphorylatable variant, CpxR_D51E_, was generated on the basis that it may cause a constitutively ‘locked-on’ CpxR phenotype, as is often the case for related RRs [44-46]. In parallel, we deleted *in silico* predicted structural elements of CpxR including the putative N-terminal-located coiled-coil domain (CpxR_Δ11–24_), a C-terminal-located wHTH domain (CpxR_Δ188–209_) as well as the flexible internal linker that separates the N- and C-terminal CpxR domains (CpxR_Δ117–132_) [43]. Again, the same CpxA_1-458_-T18 bait construct was used in combination with the newly generated prey constructs T25-CpxR_D51A_, T25-CpxR_D51E_, T25-CpxR_Δ11–24_, T25-CpxR_Δ117–132_ and T25-CpxR_Δ188–209_. Enzymatic assays revealed that CpxR could interact well with CpxA, but only if its N-terminus remained intact, such as with the fully functional CpxR_D51E_ variant (~14.7 fold increase in reporter activity) or the CpxR_Δ188–209_ variant (~16.8 fold) (Figure 2). Conversely, disruption of CpxR N-terminus, as occurred with the CpxR_D51A_, CpxR_Δ11–24_ and CpxR_Δ117–132_ variants, only permitted low reporter activity in these fusion-bearing strains (Figure 2). Although western blotting could not determine whether any of these T25-CpxR fusions were actually produced and stable, these data corroborate earlier *in silico* predictions that the CpxR N-terminus is probably involved in crosstalk with CpxA [47,48]. They also suggest that BACTH is a valid approach to further investigate this aspect of Cpx pathway activation.

### The extreme C-terminus of CpxA is dispensable for interactions with CpxR

**Figure 3:**
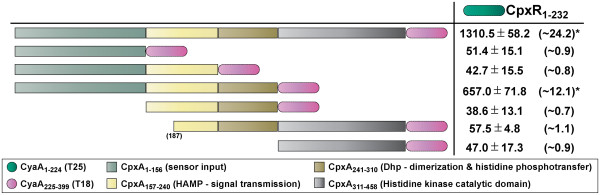
**Regions of CpxA interacting with CpxR as monitored by BACTH analysis.** Full-length CpxR_1-232_ was translationally fused to the C-terminus of CyaA_1-224_ (T25 – dark green shade) creating a T25-CpxR_1-232_ hybrid used as the ‘bait’. As in Figure 1, the same seven CpxA variants translationally fused to the N-terminus of CyaA_225-399_ (T18 – magenta shade) were used as ‘prey’ hybrids, that is CpxA_1-458_-T18, CpxA_1-156_-T18, CpxA_1-240_-T18, CpxA_1-310_-T18, CpxA_157-310_-T18, CpxA_187-458_-T18 and CpxA_311-458_-T18. BACTH interaction analysis of ‘bait’ and ‘prey’ hybrids was quantified via measurement of β-galactosidase activity and is represented as units/mg dry weight of host *E. coli* BTH101 bacteria (left column; black font). The internal positive control based upon the constructs expressing T18-Zip and T25-Zip yielded 1778.1 ± 120.3 units of β-galactosidase activity/mg dry weight of bacteria. This was ~32.8 fold more enzymatic activity produced compared to bacteria co-expressing only T18 and T25 (54.2 ± 9.0 units of β-galactosidase activity). The fold change in enzymatic activity caused by CpxR-CpxA interactions relative to this negative control is indicated in parentheses to the right. The asterisks (*) indicates a positive interaction. Data is presented as the mean (± standard error of the mean) of at least four independent experiments performed in triplicate.

A reciprocal experiment was next conducted in an effort to identify the region of CpxA necessary for engaging CpxR. We selected the T25-CpxR_1-232_ construct as bait, since it induced high reporter activity in a strain co-expressing CpxA_1-458_-T18 (see Figure 2). We also used the same prey constructs incorporating the CpxA truncated variants fused to the N-terminus of T18 and described in Figure 1. *E. coli* BTH101 expressing both bait T25-CpxR_1-232_ and prey CpxA_1-458_-T18 hybrids produced β-galactosidase levels that were ~24.2 fold higher than the negative control, and only slightly lower than the T25-Zip/T18-Zip positive control (~32.8 fold elevation) (Figure 3, data not shown). The CpxA_1-310_-T18 hybrid could also interact with bait T25-CpxR_1-232_ as indicated by ~12.1 fold higher reporter activity in the host strain (Figure 3). No remaining fusion combinations induced any significant reporter activity in BTH101. Once again, we could not confirm by western blotting whether this negligible reporter activity was due to poor interactions or unstable CpxA_1-156_-T18, CpxA_1-240_-T18, CpxA_157-310_-T18, CpxA_187-458_-T18 and CpxA_311-458_-T18 protein (data not shown). However, since the CpxA_157-310_-T18 and CpxA_187-458_-T18 fusions must at least be modestly produced (see Figure 1), this data suggests that the DHp domain in cooperation with additional upstream N-terminal CpxA sequence might be sufficient for engaging CpxR, corroborating reports focused on other SK-RR cognate pairs [47-49]. It also suggests that sequence in the extreme C-terminus of CpxA is probably not required for interacting with CpxR, at least via the BACTH assay. 

### BACTH analysis of CpxR-CpxR interactions

**Figure 4:**
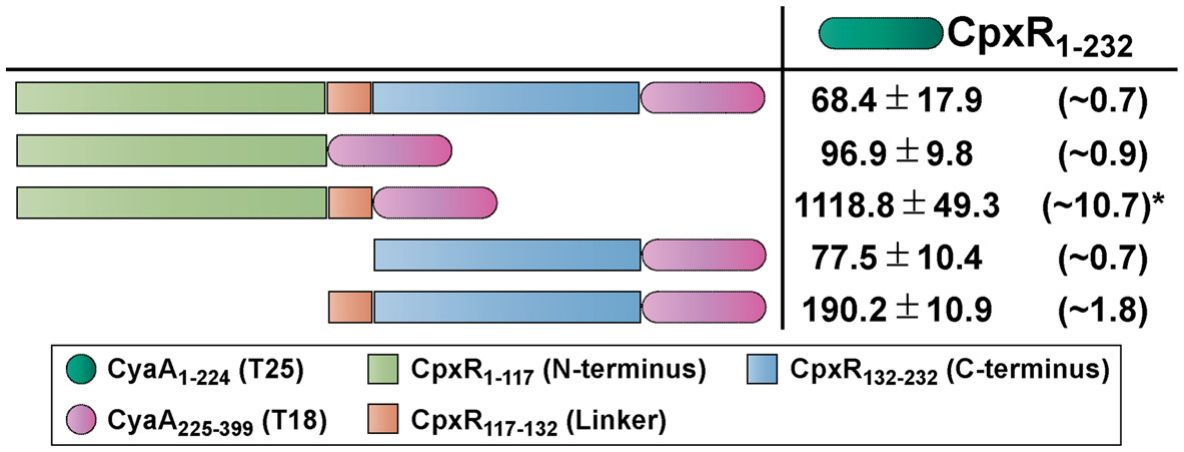
**BACTH analysis of CpxR-CpxR interactions.** Full-length CpxR_1-232_ was translationally fused to the C-terminus of CyaA_1-224_ (T25 – dark green shade) creating a T25-CpxR_1-232_ hybrid used as the ‘bait’. Full-length CpxR_1-232_ was translationally fused to the N-terminus of CyaA_225-399_ (T18 – magenta shade) giving rise to a CpxR_1-232_-T18 ‘prey’ hybrid. Based upon divisions of CpxR into N-terminal (pistachio green), internal linker (orange) and C-terminal (sky blue) domains, additional ‘prey’ T18 hybrids were constructed that consisted of only the N-terminus without linker (CpxR_1-117_-T18) or with linker (CpxR_1-132_-T18) and the C-terminus without linker (CpxR_132-232_-T18) or with linker (CpxR_117-232_-T18). BACTH interaction analysis of ‘bait’ and ‘prey’ hybrids was quantified via measurement of β-galactosidase activity and is represented as units/mg dry weight of host *E. coli* BTH101 bacteria (left column; black font). The internal positive control based upon the constructs expressing T18-Zip and T25-Zip yielded 1521.9 ± 150.6 units of β-galactosidase activity/mg dry weight of bacteria. This has ~14.6 fold more enzymatic activity than bacteria co-expressing only T18 and T25 (104.6 ± 12.9 units of β-galactosidase activity). The fold change in enzymatic activity caused by CpxR-CpxR interactions relative to this negative control is indicated in parentheses to the right. The asterisks (*) indicates a positive interaction. Data is presented as the mean (± standard error of the mean) of at least four independent experiments performed in triplicate.

Next, we explored the suitability of the BACTH assay for dissecting CpxR dimerization [50]. In the first instance, a ‘bait’ construct consisting of full-length CpxR fused to the C-terminus of T25 (T25-CpxR_1-232_) was transformed with a ‘prey’ construct of full-length CpxR fused to the N-terminus of T18 (CpxR_1-232_-T18). However, the detected β-galactosidase activity barely increased above the negative control value (Figure 4). As with all our other BACTH analysis, neither fusion was detected by western blotting (data not shown) making it difficult to conclude whether the low reporter activity represents the absence of an interaction or merely unstable protein. Curiously, co-expression of full-length CpxR fusions still did not stimulate transcription of the *lacZ* reporter even after the orientation of the T18 and T25 fusions were swapped (N-terminus vs C-terminus) (data not shown). 

Given a report that the C-terminal domain effector activity of *E. coli* CpxR can be unlocked by physically removing the N-terminal regulatory domain [43], we entertained the notion that our full-length CpxR fusions might be locked in a static structural confirmation that prevents measurable dimerization. To investigate this, we employed a series of prey constructs with T18 N-terminally fused to truncated CpxR derivatives composed of only the N-terminus without (CpxR_1-117_-T18) or with internal linker (CpxR_1-132_-T18) and the C-terminus without internal linker (CpxR_132-232_-T18) or with internal linker (CpxR_117-232_-T18). These constructs were related to those already described in Figure 2 and are also utilized in subsequent aspects of this study. These truncated CpxR-T18 prey constructs were transformed into *E. coli* BTH101 containing the full-length T25-CpxR_1-232_ bait construct. On the basis of a ~10.7 fold elevation from baseline β-galactosidase activity, T25-CpxR_1-232_ formed a strong interaction with CpxR_1-132_-T18 (Figure 4). On this occasion, the strength of this union mirrored the well established dimerization of the Zip protein used as a control (~14.6 fold elevation from baseline β-galactosidase activity) (Figure 4, data not shown). In contrast, no other interaction of truncated CpxR with full-length CpxR could be detected, even after the orientation (N-terminus vs C-terminus) or combination (‘bait’ vs ‘prey’) of T18 and T25 domains were altered (Figure 4, data not shown). This also means that full-length CpxR-CpxR_1-132_ interaction observed in the BACTH assay is conditional, being heavily influenced by the orientation and combination of the T18 and T25 fusions.

### N-terminal mediated CpxR dimerization

**Figure 5:**
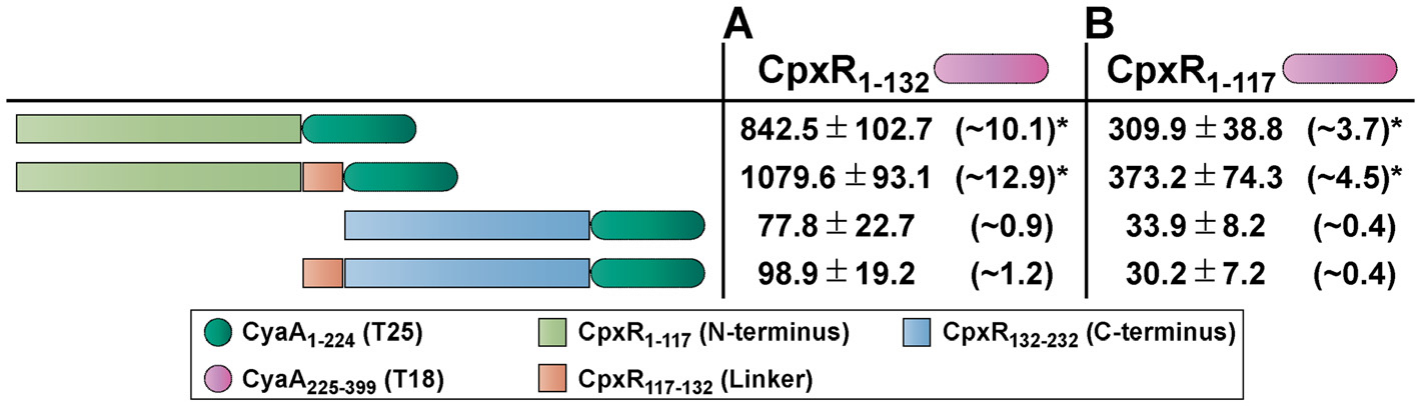
**N-terminal CpxR dimerization in BACTH assays is enhanced by inclusion of the internal CpxR linker.** The N-terminal domain of CpxR either with linker (**A**) or without linker (**B**) were translationally fused to the N-terminus of CyaA_225-399_ (T18 – magenta shade) creating the ‘bait’ CpxR_1-132_-T18 and CpxR_1-117_-T18 hybrids respectively. As ‘prey’ hybrids, the CpxR N-terminus without linker (CpxR_1-117_-T25) or with linker (CpxR_1-132_-T25) and the C-terminus without linker (CpxR_132-232_-T25) or with linker (CpxR_117-232_-T25) were fused to the N-terminus of CyaA_1-224_ (T25 – dark green). Interactions between ‘bait’ and ‘prey’ hybrids were again quantified via measurement of β-galactosidase activity (left columns; black font). Measurement of the interaction between T18-Zip and T25-Zip yielded 1449.1 ± 113.2 units of β-galactosidase activity was ~17.3 fold more than the enzymatic activity produced by negative-control bacteria co-expressing only T18 and T25 (83.8 ± 16.3 β-galactosidase activity units). The fold change in enzymatic activity caused by CpxR-CpxR interactions relative to this negative control is indicated in parentheses to the right. The asterisks (*) indicates a positive interaction. Data is presented as the mean (± standard error of the mean) of at least four independent experiments performed in triplicate.

Having the goal to better define internal interacting regions of CpxR, additional BACTH analyses were performed based upon the pair-wise transformation of *E. coli* BTH101 using CpxR_1-132_-T18 as the bait with variable prey constructs of T25 N-terminally fused to truncated CpxR derivatives (CpxR_1-117_-T25, CpxR_1-132_-T25, CpxR_132-232_-T25 and CpxR_117-232_-T25). β-galactosidase levels were ~10.1 and ~12.9 fold higher in bacteria co-expressing CpxR_1-132_-T18/CpxR_1-117_-T25 and CpxR_1-132_-T18/CpxR_1-132_-T25 bait/prey pairs respectively (Figure 5A). This compared to the positive control that yielded β-galactosidase levels that were ~17.3 fold higher. Hence, N-terminal CpxR with linker could interact with itself or with N-terminal CpxR lacking the linker. Significantly, it did not matter if the T18 and T25 domains were switched in either orientation (N-terminus vs C-terminus) or combination (bait vs prey), the β-galactosidase levels were still elevated (data not shown). These positive interactions indicate all CpxR N-terminal fusions are stably produced. To explore whether the internal linker contributes to the interactions of the CpxR N-terminus, parallel BACTH studies with the same prey constructs as used in Figure 5A were performed with a T18 bait construct fused at the N-terminus to truncated CpxR composed of only the N-terminus without linker (CpxR_1-117_-T25). Elevated β-galactosidase activity indicated that the CpxR N-terminus alone could still interact with itself and with N-terminal CpxR containing linker (Figure 5B). Moreover, these interactions were consistently observed regardless of the T18/T25 fusion combinations used (data not shown). Consistently however, the highest reporter activity was achieved for the CpxR_1-132_-T18/CpxR_1-132_-T25 interaction pair i.e.: when the linker was present (Figure 5). These data therefore reveal that the linker is not essential for interactions of the CpxR-N terminus but may enhance them. On the other hand, we routinely observed low reporter output by strains harboring co-expressing CpxR N-terminal and C-terminal fusions (Figure 5) or between C-terminal fusions alone (data not shown). While this may be indicative that the C-terminus is not involved in CpxR dimerization *per se,* the data is inconclusive because we cannot confirm stable production of the C-terminal fusions, either by western blotting, or inferred by high reporter output from fusion-bearing strains. Nevertheless, the presented data definitively supports using the BACTH assay for studying dimerization of the CpxR N-terminus when produced in isolation.

### Key structural features of CpxR mediates homodimerization

**Figure 6:**
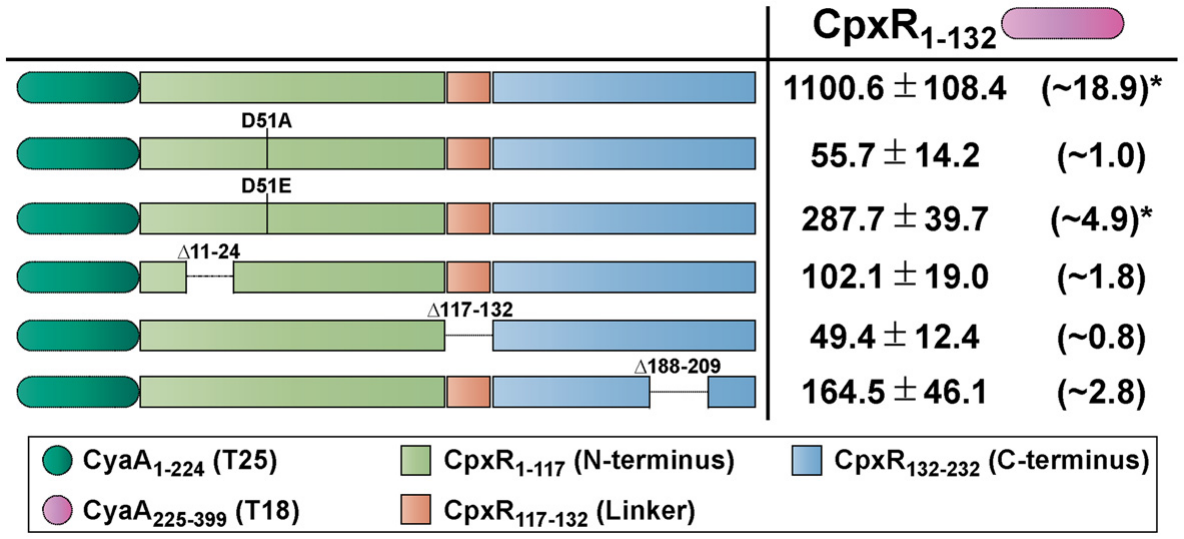
**Regions of CpxR interacting with N-terminal CpxR as monitored by BACTH analysis.** The N-terminal domain of CpxR either with linker was translationally fused to the N-terminus of CyaA_225-399_ (T18 – magenta shade) creating the ‘bait’ CpxR_1-132_-T18 hybrid. As already used in Figure 2, a selection of full-length or near full-length CpxR variants translationally fused to the C-terminus of CyaA_1-224_ (T25 – dark green shade) were used as ‘prey’ hybrids; that is T25-CpxR_D51A_ and T25-CpxR_D51E_ lacking the phosphorylated aspartate residue as well as T25-CpxR_Δ11–24_, T25-CpxR_Δ117–132_ and T25-CpxR_Δ188–209_ lacking the putative N-terminal coiled-coil, internal linker and C-terminal winged helix-turn-helix regions respectively. We also employed the T25-CpxR_1-232_ hybrid containing wild type CpxR sequence. BACTH interaction analysis of ‘bait’ and ‘prey’ hybrids was quantified via measurement of β-galactosidase activity and is represented as units/mg dry weight of host *E. coli* BTH101 bacteria (left column; black font). The internal positive control based upon constructs expressing T18-Zip and T25-Zip yielded 1495.1 ± 237.3 units of β-galactosidase activity/mg dry weight of bacteria. This was on average ~25.6 fold more enzymatic activity produced compared to bacteria co-expressing only T18 and T25 (58.3 ± 14.1 units of β-galactosidase activity). The fold change in enzymatic activity caused by CpxR-CpxR interactions relative to this negative control is indicated in parentheses to the right. The asterisks (*) indicates a positive interaction. Data is presented as the mean (± standard error of the mean) of at least four independent experiments performed in triplicate.

Since the CpxR N-terminus dimerizes with full-length CpxR (Figure 4), we designed another experiment in an effort to highlight structural regions within the full-length CpxR that may mediate specific interactions with the N-terminal region. As in Figure 4 and 5, CpxR_1-132_-T18 was used as the ‘bait’ fusion. Additionally, we capitalized on the availability of variable ‘prey’ constructs previously used in Figure 2, which were composed of T25 fused to the N-terminal end of the larger CpxR constructs giving rise to T25-CpxR_D51A_, T25-CpxR_D51E_, T25-CpxR_Δ11–24_, T25-CpxR_Δ117–132_ and T25-CpxR_Δ188–209_. Native full-length CpxR fused to T25 (T25-CpxR_1-232_) served as a control. In *E. coli* BTH101, the interacting hybrids CpxR_1-132_-T18 and T25-CpxR_1-232_ induced reporter activity that was ~18.9 fold higher than background (Figure 6). Only one other bait/prey combination – CpxR_1-132_-T18 with the potentially ‘locked-on’ CpxR_D51E_ variant mimicking a phosphorylated state (T25-CpxR_D51E_) – induced higher reporter activity of ~4.9 fold (Figure 6). Although we are unable to confirm the stable production of the non-phosphorylated and inactive CpxR_D51A_ variant (T25-CpxR_D51A_), the low reporter activity of this fusion-bearing strain could indicate that interactions with N-terminal and full-length CpxR derivates requires phosphorylation at position D_51_. The ability to interact was also lost upon generating Δ11-24, Δ117-132 and Δ188-209 deletions in *cpxR.* Hence, dimerization might also require CpxR to be inherently flexible, garnered by structural and/or functional elements encoded along the protein’s entire length. At least one of these regions encompassing residues 188 to 209 might be one such segment, since this fusion must be capable of being stably produced (see Figure 2).

### Homodimerization of CpxR as determined by reconstitution of the λcI repressor

**Figure 7:**
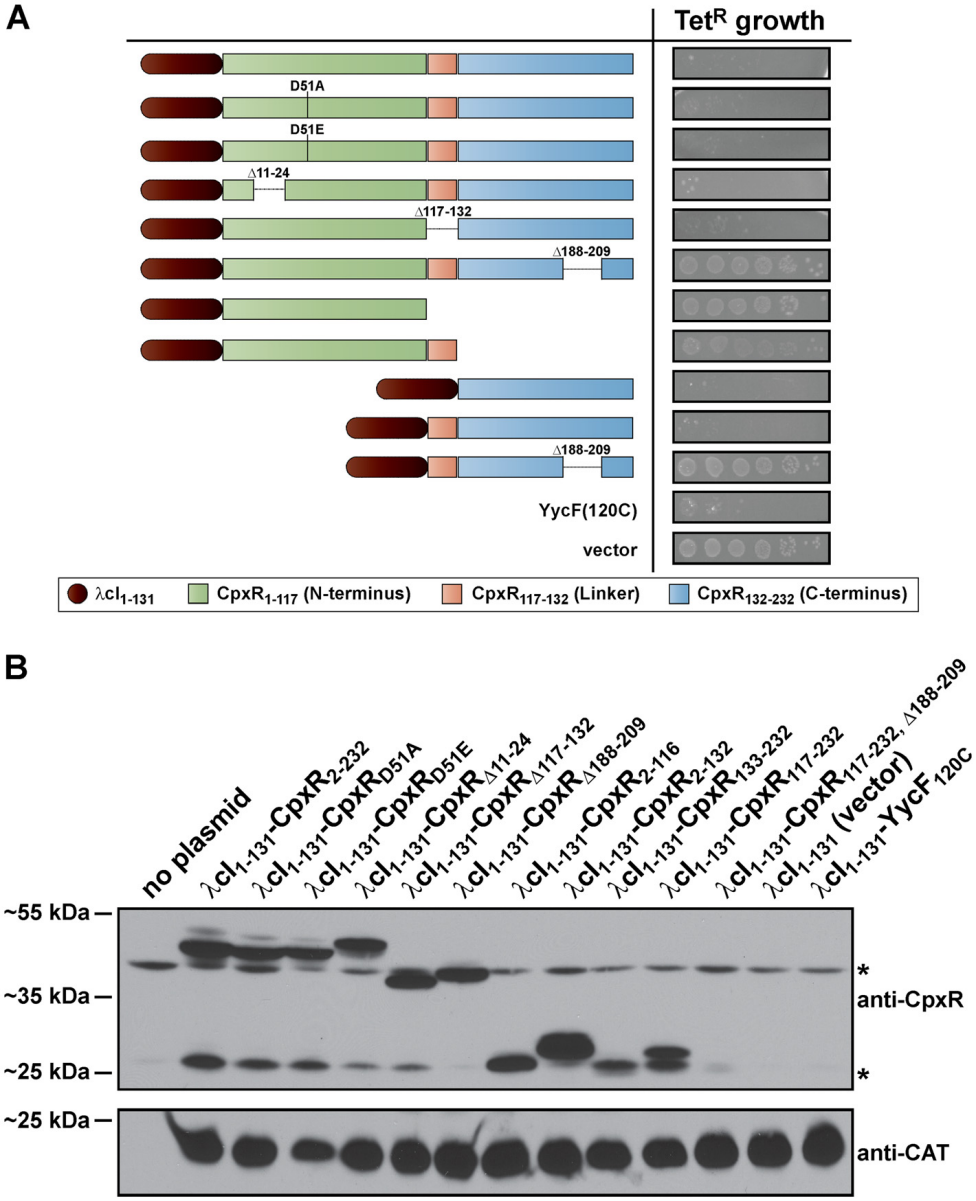
**Reconstitution of the λcI repressor function through CpxR homodimerization.** Fusion of CpxR_n_ variants to the C-terminus of λcI_1-131_ (auburn brown shade) were generated. When expressed in *E. coli* JM109, growth was assessed by spotting 5 μl of 10-fold serially diluted (10^0^, 10^−1^, 10^−2^, 10^−3^, 10^−4^ and 10^−5^) exponentially grown cultures onto LB agar containing 10 μg/ml tetracycline (Tet) and 0.1 mM IPTG (**A**). The assay was controlled through the expression of inactive λcI_1-131_ alone [vector; pKWY2428] or the dimerization-competent fusion λcI_1-131_-YycF_120-235_ [YycF(120C); pKWY-YycF(120C)] [36]. Protein lysates were also fractionated on 12% acrylamide SDS-PAGE and analysed by western blotting (**B**). Fusions of λcI_1-131_ to the N-terminus of CpxR_n_ variants were detected with rabbit polyclonal anti-CpxR antiserum. Samples were also probed with antiserum raised in rabbit and specific for chloramphenicol acetyltransferase (anti-CAT) to confirm the loading of an equal quantity of protein in each lane. The asterisks (*) highlight unknown protein bands that cross-react non-specifically with antibodies in the sera or, in some cases may represent a degradation product of the recombinant fusion proteins. Shown to the left is the approximate mobility of molecular weight standards (PageRuler^TM^ Plus Prestained Protein Ladder, Thermo Scientific).

In parallel, we also employed a homodimerization assay based upon the reconstitution of the Lamda cI repressor (λcI) protein. This assay was effective in studying homodimerization of the YycG SK from *Staphylococcus aureus*[51] and the YycF RR from *B. subtilis*[36]. It is also an attractive basis for developing a high throughput screening system for the discovery of novel anti-bacterials targeting homodimerization of TCRS constituents [51]. Various alleles coding for different CpxR derivatives were translationally fused to the C-terminus of the first 131 residues of λcI (λcI_1-131_-CpxR_n_) expressed from plasmid pKWY2428 [36]. Reconstitution of an active repressor dimer can occur through the homodimerization of CpxR. In these situations where the fused CpxR protein does homodimerize, reconstituition of λcI_1-131_ DNA binding activity specifically represses transcription from the *tet* resistance gene promoter present on the λcI_1-131_-containing plasmid. This confers tetracycline sensitivity to the host bacterial strain *E. coli* JM109 [36]. An absence of homodimerization however will confer tetracycline resistance (Tet^R^) to the host strain. Contrary to our BACTH analysis, full-length CpxR (λcI_1-131_-CpxR_2-232_) and the two C-terminal CpxR variants with linker (λcI_1-131_-CpxR_117-232_) or without linker (λcI_1-131_-CpxR_133-232_) were all able to homodimerize as determined by heightened sensitivity of JM109 (reduced growth) on tetracycline supplemented LB agar (Figure 7A). The extent of this growth restriction mirrored that achieved for the RR YycF C-terminal region encompassing residues 120 to 235 fused to λcI_1-131_ and expressed from the plasmid pKWY-YycF(120C) [36] (Figure 7A). Interestingly, dimerization and growth suppression by the λcI_1-131_-CpxR_133-232_ fusion occurred despite steady-state protein levels being quite minimal (Figure 7B). In further contradiction to our BACTH analysis (see Figure 5), we were unable to observe any growth restriction of JM109 when expressing λcI_1-131_-CpxR_2-116_ or λcI_1-131_-CpxR_2-132_ (Figure 7A), even though protein accumulated to high levels (especially in the case of λcI_1-131_-CpxR_2-132_) (Figure 7B). This extensive growth was consistent with the negative control expressing only λcI_1-131_ from the plasmid pKWY2428 [36] (Figure 7A). Hence, N-terminal CpxR with or without the internal linker could not homodimerize when fused at the C-terminus of λcI_1-131_, although independent fusions to T25 and T18 domains still support homodimerization (see Figure 5 and 6). Thus, the λcI homodimerization assay is a tool especially suitable for studying dimerization of the CpxR C-terminus when produced in isolation. 

To further explore λcI_1-131_-CpxR homodimerization, we analyzed essentially full-length CpxR derivatives carrying the specific deletions of the N-terminal coiled-coil (λcI_1-131_-CpxR_Δ11–24_), internal linker (λcI_1-131_-CpxR_Δ117–132_) and C-terminal wHTH domain (λcI_1-131_-CpxR_Δ188–209_). In addition, we examined fusions harboring site-directed mutations in the N-terminal phosphorylation site (λcI_1-131_-CpxR_D51A_ and λcI_1-131_-CpxR_D51E_). Importantly, we could confirm that all of these fusion proteins accumulated in the cytoplasm of JM109 cells (Figure 7B). Clearly however, CpxR_188-209_ exhibits a major defect in dimerization capacity (Figure 7A); a finding also observed in the BACTH assay (see Figure 6). This dimerization defect was also conferred to the fusion expressing only the C-terminus (λcI_1-131_-CpxR_117-232, Δ188–209_) (Figure 7A), although this is less conclusive because this fusion was poorly expressed (Figure 7B). On the other hand, non-phosphorylated CpxR or CpxR lacking the N-terminal coiled-coil domain or internal linker, could all still homodimerize (Figure 7A). Once again, this data is at odds with our BACTH analysis, where a CpxR_D51A_, CpxR_Δ11–24_ or a CpxR_Δ117–132_ mutation inhibited subsequent CpxR interactions (see Figure 6).

## Discussion

TCRSs play a significant role in the regulation of physiological processes in the bacterial cell ranging from the control of single genes to diverse multi-cellular behavior. This occurs by means of transcriptional, post transcriptional and post translational regulation whereby TCRSs exploit a variety of protein-protein and protein-DNA interactions [3,42,52]. In this study, we attempted to gain some insight into the protein-protein interactions that are the hallmark of CpxA-CpxR signal transduction.

BACTH analysis revealed that full-length CpxR and the truncated N-terminal derivatives were able to interact together, albeit in a manner conditionally dependent upon the direction and orientation of the T18 and T25 fusions. Interactions of full-length CpxR were also observed in our λcI homodimerization analysis, but we were unable to validate dimerization of N-terminal truncates via this method. Interactions dependent upon the positioning of the fused CyaA domains might well indicate that the CpxR-CpxR interaction occurs as a head-to-head symmetrical dimer mediated by a N-terminal α_4_-β_5_-α_5_ interface highly conserved among OmpR-PhoB RR family members [30,42]. Indeed, structural studies of full-length and isolated N-terminal receiver domains of various RRs belonging to this OmpR-PhoB family suggest head-to-head dimerization is common and functionally important [53-56]. Hence, further studies are required to better understand how the α_4_-β_5_-α_5_ interface of CpxR determines CpxR dimerization orientation and contributes to subsequent DNA binding and gene transcription control.

Use of the λcI assay indicated that the CpxR C-terminus could readily dimerize; a finding not observed during our BACTH analysis. Nevertheless, a previous study has demonstrated that solitary *trans-*expression of C-terminal CpxR is constitutively active and capable of transcriptional control of an array of target genes in *E. coli*[43]. Moreover, the degree of CpxR activation was independent of the internal linker [43]. This data was explained through an expectation that the C-terminal effector domain can alone dimerize to activate its target genes; a notion we can now experimentally verify via λcI homodimerization analysis. Incidentally, this analysis also demonstrated that the linker was not required for this dimerization process as was previously assumed [43]. In addition to the ability to interact with itself, constitutive activation of the CpxR C-terminus implies that this region must engage one of the six subunits of the RNA polymerase holoenzyme in order to specifically modify its transcription output from targeted genes. Direct interaction with the RNA polymerase through the C-terminus is well established for other RRs of the wHTH family; prototype OmpR interacts with the α subunit [57-63] and PhoB the σ^70^ subunit [64-66]. Hence, it is of considerable interest to determine how the C terminus of CpxR coordinates dimerization, DNA binding and modulation of RNA polymerase activity in the pathogenic Yersiniae.

The studies performed to elucidate the role of the phospho-modified residue (D51) were intriguing. The non-phosphorylated and functionally inactive CpxR_D51A_ variant could not interact with CpxA or other CpxR variants via BACTH analysis. The caveat here concerns our inability to confirm stable production of CpxR_D51A_ fused to either T18 or T25. Where production was confirmed in the λcI homodimerization assay, λcI_1-131_-CpxR_D51A_ did dimerize. Clearly therefore, this aspect requires more investigation, especially given the suggestion that phosphorylation promotes homodimer formation within the OmpR/PhoB subfamily [50]. Intriguingly, the potentially ‘locked-on’ CpxR_D51E_ always maintained the ability to interact. This suggests that CpxR_D51A_ and CpxR_D51E_ are functionally distinct, the glutamate having the ability to mimic phosphorylation at residue 51 to maintain transcriptional activity. We have confirmed that CpxR_D51E_ does maintain function in *Y. pseudotuberculosis* (EJT *et al.,* unpublished), while purified CpxR_D51A_ is not phosphorylated *in vitro* by small phospho donors that in turn dramatically lessons target DNA binding ability [9]. It is evident therefore that conformational change induced by phosphorylation at D_51_ permits certain inter- and intra-molecular interactions. Based on these data, we anticipate that performing a thorough pair-wise molecular and biochemical characterization of the CpxR_D51A_ and CpxR_D51E_ variants will benefit our understanding of the complexities of CpxR-mediated transcriptional output mechanisms.

Our investigation into the role of the CpxR linker region (residues 117–132) in inter- and intra-molecular interactions was inconclusive. Both BACTH and λcI homodimerization studies revealed the linker to be non-essential for homodimerization of any CpxR truncated variant. However, the near full-length CpxR_Δ117–132_ variant lost the ability to engage with the CpxR N-terminus and with full-length CpxA via BACTH analysis, although it still efficiently dimerized in λcI-based studies. Hence, it seems that the linker has a function in facilitating protein-protein interactions, but this ability is context dependent. Recent studies of other members of the OmpR/PhoB subfamily are starting to shed some light on internal linker function [67-69]. Collectively, these demonstrate how linker sequence composition and length can influence RR interdomain interactions, DNA binding and subsequent transcriptional output. Presumably, the linker optimizes N- and C-terminal domain function by imparting physical separation as well as offering inherent flexibility that assists in domain conformational changes as a consequence of phosphorylation and/or DNA binding. Given this potential for influencing RR function, a detailed investigation of the CpxR linker is warranted.

The C-terminal wHTH DNA binding domain is also likely to contribute to CpxR homodimerization. While we and others have shown that truncated CpxR can dimerize even in the complete absence of the C-terminus (this study) [50], restricted removal of only residues 188 to 209 encompassing the wHTH prevents subsequent dimerization of CpxR_Δ188–209_ in both BACTH and λcI homodimerization assays. While not tested here, the role of the wHTH domain in CpxR dimer formation might well relate to the effects of binding to target DNA. After all, it is established that DNA binding by non-phosphorylated OmpR is a mechanism to enhance phosphorylation and dimerization capacity [70,71]. Hence, the wHTH motif may play a dual role in both RR target DNA binding and dimerization. On the basis of our own DNA binding assays, where non-phosphorylated native CpxR [8], but not the non-phosphorylated CpxR_D51A_ mutant [9] only very weakly bound to target DNA, it is conceivable that initial binding can dramatically enhance phosphorylation and then dimerization.

Although both the BACTH and λcI homodimerization assay were highly reproducible, they were often contradictory in their ability to predict interactions. This should not mean that these assays are unreliable or not physiologically relevant. However, it does highlight the need to perform in parallel other complementing experimental analyses to ensure relevancy of the phenotypic data. No doubt, creation of hybrid proteins fused with structurally distinct CyaA or λcI tags could diminish inherent protein flexibility or stability, curbing the subsequent ability to measure authentic protein-protein interactions. Indeed, a good correlation between protein instability and the absence of interactions in a BACTH assay has already been stated [37]. Despite our numerous attempts, we could not detect any T18 or T25 fusions, including those reliably inducing high reporter activities, such as the positive control. However, we could infer from numerous positive interaction data sets involving various T18 and T25 fusion combinations that the majority of our fusions must, to some extent, be stably produced. The notable exceptions to this deduction are the fusions involving CpxR_D51A_ and it’s C-terminal domain when in isolation. We are therefore confident that BACTH analysis is useful for CpxR N-terminal interaction studies, but it may have limited use for similar studies of the C-terminus. Critically, the majority of our λcI fusions were produced and stable. Hence, product instability cannot account for the failure of λcI_1-131_-CpxR_2-116_ and λcI_1-131_-CpxR_2-132_ fusions to homodimerize. Moreover, as homodimerization of similar CpxR N-terminal fusions occurred via BACTH analysis, poor production alone cannot explain the contradictory results using the two reporter systems. This can be further illustrated by observations with the stably produced full-length CpxR; it could dimerize to itself only when fused to λcI, but via BACTH analysis could only interact with CpxA or the CpxR N-terminus. Thus, it is apparent that individual domains appended to either CpxA_n_ or CpxR_n_ variants can alter their structural context in unique ways that significantly influence protein-protein interactions, making no one assay fully self-sufficient. By necessity therefore, future work will analyze the significance of these CpxR and CpxA interaction motifs by means of other sensitive interaction techniques in concert with analysis of pertinent *in vivo* phenotypes.

## Conclusions

We have shown here that the BACTH and λcI assays are two simple and complementary approaches amenable to identify the independent modular homo- and hetero-interactions of CpxA and CpxR. Notably, the former is ideally suited to analyze the molecular context of CpxR interactions involving the N-terminus, while the latter can aid in dissecting interactions of the CpxR C-terminus. Moreover, when combined with *in vivo* assays designed to assess bacterial survival and virulence, a better understanding of the roles of the CpxA-CpxR TCRS in *Y. pseudotuberculosis* will ensue.

## Abbreviations

ECS: Extracytoplasmic stress; Cpx: Conjugative pilus expression; RR: Response regulator; SK: Sensor kinase; TCRS: Two-component regulatory system; wHTH: winged helix-turn-helix; LB: Luria-Bertani; IPTG: Isopropyl-β-d-thiogalactopyranoside; X-Gal: 5-Bromo-4-chloro-3-indolyl-β-d-galactopyranoside; Ap: Ampicillin; Km: Kanamycin; Cm: Chloramphenicol; Tc: Tetracycline, BACTH, bacterial adenylate cyclase two-hybrid; CAP: Catabolite activator protein; cAMP: Cyclic adenosine monophosphate.

## Competing interests

The authors declare that they have no competing interests.

## Authors’ contributions

ET and JM participated in study design, its coordination and performed all experimentation. MF conceived of the study, and participated in its design and coordination and drafted the manuscript. All authors analyzed the data and read and approved the final manuscript.

## Supplementary Material

Additional file 1 **Table S1.** Bacteria strains and plasmids used in this study. Click here for file

Additional file 2 **Table S2.** Oligonucleotides used in this study. Click here for file

## References

[B1] Hunke S Keller R Muller VS Signal integration by the Cpx-envelope stress system FEMS Microbiol Lett 2012 326 12 22 10.1111/j.1574-6968.2011.02436.x 22092888

[B2] Vogt SL Raivio TL Just scratching the surface: an expanding view of the Cpx envelope stress response FEMS Microbiol Lett 2012 326 2 11 10.1111/j.1574-6968.2011.02406.x 22092948

[B3] Krell T Lacal J Busch A Silva-Jimenez H Guazzaroni ME Ramos JL Bacterial sensor kinases: diversity in the recognition of environmental signals Annu Rev Microbiol 2010 64 539 59 10.1146/annurev.micro.112408.134054 20825354

[B4] Itou H Tanaka I The OmpR-family of proteins: insight into the tertiary structure and functions of two-component regulator proteins J Biochem 2001 129 343 50 10.1093/oxfordjournals.jbchem.a002863 11226872

[B5] Labandeira-Rey M Brautigam CA Hansen EJ Characterization of the CpxRA regulon in Haemophilus ducreyi Infect Immun 2010 78 4779 91 10.1128/IAI.00678-10 20805330 PMC2976327

[B6] Bury-Mone S Nomane Y Reymond N Barbet R Jacquet E Imbeaud S Jacq A Bouloc P Global analysis of extracytoplasmic stress signaling in Escherichia coli PLoS Genet 2009 5 e1000651 10.1371/journal.pgen.1000651 19763168 PMC2731931

[B7] Carlsson KE Liu J Edqvist PJ Francis MS Extracytoplasmic-stress-responsive pathways modulate type III secretion in Yersinia pseudotuberculosis Infect Immun 2007 75 3913 24 10.1128/IAI.01346-06 17517869 PMC1951977

[B8] Carlsson KE Liu J Edqvist PJ Francis MS Influence of the Cpx extracytoplasmic-stress-responsive pathway on Yersinia sp.-eukaryotic cell contact Infect Immun 2007 75 4386 4399 10.1128/IAI.01450-06 17620356 PMC1951158

[B9] Liu J Obi IR Thanikkal EJ Kieselbach T Francis MS Phosphorylated CpxR restricts production of the RovA global regulator in Yersinia pseudotuberculosis PLoS One 2011 6 e23314 10.1371/journal.pone.0023314 21876746 PMC3158067

[B10] Liu J Thanikkal EJ Obi IR Francis MS Elevated CpxR approximately P levels repress the Ysc-Yop type III secretion system of Yersinia pseudotuberculosis Res Microbiol 2012 http://dx.doi.org/10.1016/j.resmic.2012.07.010 10.1016/j.resmic.2012.07.010 22842077

[B11] Cathelyn JS Crosby SD Lathem WW Goldman WE Miller VL RovA, a global regulator of Yersinia pestis, specifically required for bubonic plague 2006 103 13514 9 10.1073/pnas.0603456103 PMC1569194 16938880

[B12] Yang F Ke Y Tan Y Bi Y Shi Q Yang H Qiu J Wang X Guo Z Ling H Cell membrane is impaired, accompanied by enhanced type III secretion system expression in Yersinia pestis deficient in RovA regulator PLoS One 2010 5 e12840 10.1371/journal.pone.0012840 20862262 PMC2941471

[B13] Cathelyn JS Ellison DW Hinchliffe SJ Wren BW Miller VL The RovA regulons of Yersinia enterocolitica and Yersinia pestis are distinct: evidence that many RovA-regulated genes were acquired more recently than the core genome Mol Microbiol 2007 66 189 205 10.1111/j.1365-2958.2007.05907.x 17784909

[B14] Francis MS Kidd PP Secretion systems and metabolism in the pathogenic Yersiniae Stress response in pathogenic bacteria 2011 CABI Publishing, Wallingford, Oxfordshire, United Kingdom 185 220

[B15] Dorel C Lejeune P Rodrigue A The Cpx system of Escherichia coli, a strategic signaling pathway for confronting adverse conditions and for settling biofilm communities? Res Microbiol 2006 157 306 14 10.1016/j.resmic.2005.12.003 16487683

[B16] Raivio TL Envelope stress responses and Gram-negative bacterial pathogenesis Mol Microbiol 2005 56 1119 28 10.1111/j.1365-2958.2005.04625.x 15882407

[B17] Rowley G Spector M Kormanec J Roberts M Pushing the envelope: extracytoplasmic stress responses in bacterial pathogens Nat Rev Microbiol 2006 4 383 94 10.1038/nrmicro1394 16715050

[B18] Kwon E Kim DY Ngo TD Gross CA Gross JD Kim KK The crystal structure of the periplasmic domain of Vibrio parahaemolyticus CpxA Protein Sci 2012 21 1334 43 10.1002/pro.2120 22760860 PMC3631362

[B19] Casino P Rubio V Marina A The mechanism of signal transduction by two-component systems Curr Opin Struct Biol 2010 20 763 71 10.1016/j.sbi.2010.09.010 20951027

[B20] Aravind L Ponting CP The cytoplasmic helical linker domain of receptor histidine kinase and methyl-accepting proteins is common to many prokaryotic signalling proteins FEMS Microbiol Lett 1999 176 111 6 10.1111/j.1574-6968.1999.tb13650.x 10418137

[B21] Williams SB Stewart V Functional similarities among two-component sensors and methyl-accepting chemotaxis proteins suggest a role for linker region amphipathic helices in transmembrane signal transduction Mol Microbiol 1999 33 1093 102 10510225 10.1046/j.1365-2958.1999.01562.x

[B22] Wolanin PM Thomason PA Stock JB Histidine protein kinases: key signal transducers outside the animal kingdom Genome Biol 2002 3 reviews3013.1–3013.8 10.1186/gb-2002-3-10-reviews3013 PMC244915 12372152

[B23] Stewart RC Protein histidine kinases: assembly of active sites and their regulation in signaling pathways Curr Opin Microbiol 2010 13 133 41 10.1016/j.mib.2009.12.013 20117042 PMC2847664

[B24] Dutta R Qin L Inouye M Histidine kinases: diversity of domain organization Mol Microbiol 1999 34 633 40 10.1046/j.1365-2958.1999.01646.x 10564504

[B25] Perego M Hoch JA Protein aspartate phosphatases control the output of two-component signal transduction systems Trends Genet 1996 12 97 101 10.1016/0168-9525(96)81420-X 8868347

[B26] Kenney LJ How important is the phosphatase activity of sensor kinases? Curr Opin Microbiol 2010 13 168 76 10.1016/j.mib.2010.01.013 20223700 PMC2853374

[B27] Marina A Mott C Auyzenberg A Hendrickson WA Waldburger CD Structural and mutational analysis of the PhoQ histidine kinase catalytic domain. Insight into the reaction mechanism J Biol Chem 2001 276 41182 41190 10.1074/jbc.M106080200 11493605

[B28] Bilwes AM Quezada CM Croal LR Crane BR Simon MI Nucleotide binding by the histidine kinase CheA Nat Struct Biol 2001 8 353 60 10.1038/86243 11276258

[B29] Gao R Stock AM Molecular strategies for phosphorylation-mediated regulation of response regulator activity Curr Opin Microbiol 2010 13 160 7 10.1016/j.mib.2009.12.009 20080056 PMC2859964

[B30] Bourret RB Receiver domain structure and function in response regulator proteins Curr Opin Microbiol 2010 13 142 9 10.1016/j.mib.2010.01.015 20211578 PMC2847656

[B31] Gotoh Y Eguchi Y Watanabe T Okamoto S Doi A Utsumi R Two-component signal transduction as potential drug targets in pathogenic bacteria Curr Opin Microbiol 2010 13 232 239 10.1016/j.mib.2010.01.008 20138000

[B32] French GL The continuing crisis in antibiotic resistance Int J Antimicrob Agents 2010 36 Suppl 3 S3 7 21129629 10.1016/S0924-8579(10)70003-0

[B33] Hanahan D Glover DM Techniques for transformation of E. coli DNA Cloning. A practical approach 1985 IRL Press Ltd, Oxford, United Kingdom 109 136

[B34] Karimova G Pidoux J Ullmann A Ladant D A bacterial two-hybrid system based on a reconstituted signal transduction pathway 1998 95 5752 6 10.1073/pnas.95.10.5752 PMC20451 9576956

[B35] Karimova G Dautin N Ladant D Interaction network among Escherichia coli membrane proteins involved in cell division as revealed by bacterial two-hybrid analysis J Bacteriol 2005 187 2233 43 10.1128/JB.187.7.2233-2243.2005 15774864 PMC1065216

[B36] Watanabe T Hashimoto Y Umemoto Y Tatebe D Furuta E Fukamizo T Yamamoto K Utsumi R Molecular characterization of the essential response regulator protein YycF in Bacillus subtilis J Mol Microbiol Biotechnol 2003 6 155 63 10.1159/000077246 15153768

[B37] Workentine ML Chang L Ceri H Turner RJ The GacS-GacA two-component regulatory system ofPseudomonas fluorescens: a bacterial two-hybrid analysis. FEMS Microbiol Lett 2009 292 50 6 10.1111/j.1574-6968.2008.01445.x 19191877

[B38] Scheu PD Witan J Rauschmeier M Graf S Liao YF Ebert-Jung A Basche T Erker W Unden G The CitA/CitB two-component system regulating citrate fermentation in Escherichia coli and its relation to the DcuS/DcuR system in vivo J Bacteriol 2012 194 636 645 10.1128/JB.06345-11 22101843 PMC3264064

[B39] Jovanovic G Engl C Buck M Physical, functional and conditional interactions between ArcAB and phage shock proteins upon secretin-induced stress in Escherichia coli Mol Microbiol 2009 74 16 28 10.1111/j.1365-2958.2009.06809.x 19682256 PMC2764110

[B40] Luttmann D Heermann R Zimmer B Hillmann A Rampp IS Jung K Gorke B Stimulation of the potassium sensor KdpD kinase activity by interaction with the phosphotransferase protein IIA(Ntr) in Escherichia coli Mol Microbiol 2009 72 978 94 10.1111/j.1365-2958.2009.06704.x 19400808

[B41] Gerken H Charlson ES Cicirelli EM Kenney LJ Misra R MzrA: a novel modulator of the EnvZ/OmpR two-component regulon Mol Microbiol 2009 72 1408 22 10.1111/j.1365-2958.2009.06728.x 19432797 PMC2727453

[B42] Gao R Stock AM Biological insights from structures of two-component proteins Annu Rev Microbiol 2009 63 133 54 10.1146/annurev.micro.091208.073214 19575571 PMC3645274

[B43] Tapparel C Monod A Kelley WL The DNA-binding domain of the Escherichia coli CpxR two-component response regulator is constitutively active and cannot be fully attenuated by fused adjacent heterologous regulatory domains Microbiology 2006 152 431 41 10.1099/mic.0.28538-0 16436431

[B44] Klose KE Weiss DS Kustu S Glutamate at the site of phosphorylation of nitrogen-regulatory protein NTRC mimics aspartyl-phosphate and activates the protein J Mol Biol 1993 232 67 78 10.1006/jmbi.1993.1370 8331671

[B45] Lan CY Igo MM Differential expression of the OmpF and OmpC porin proteins in Escherichia coli K-12 depends upon the level of active OmpR J Bacteriol 1998 180 171 4 9422609 10.1128/jb.180.1.171-174.1998 PMC106865

[B46] Domian IJ Quon KC Shapiro L Cell type-specific phosphorylation and proteolysis of a transcriptional regulator controls the G1-to-S transition in a bacterial cell cycle Cell 1997 90 415 24 10.1016/S0092-8674(00)80502-4 9267022

[B47] Skerker JM Perchuk BS Siryaporn A Lubin EA Ashenberg O Goulian M Laub MT Rewiring the specificity of two-component signal transduction systems Cell 2008 133 1043 54 10.1016/j.cell.2008.04.040 18555780 PMC2453690

[B48] Szurmant H Hoch JA Interaction fidelity in two-component signaling Curr Opin Microbiol 2010 13 190 7 10.1016/j.mib.2010.01.007 20133181 PMC2847666

[B49] Siryaporn A Perchuk BS Laub MT Goulian M Evolving a robust signal transduction pathway from weak cross-talk Mol Syst Biol 2010 6 452 21179024 10.1038/msb.2010.105 PMC3018164

[B50] Gao R Tao Y Stock AM System-level mapping of Escherichia coli response regulator dimerization with FRET hybrids Mol Microbiol 2008 69 1358 72 10.1111/j.1365-2958.2008.06355.x 18631241 PMC2586830

[B51] Furuta E Yamamoto K Tatebe D Watabe K Kitayama T Utsumi R Targeting protein homodimerization: a novel drug discovery system FEBS Lett 2005 579 2065 70 10.1016/j.febslet.2005.02.056 15811319

[B52] Galperin MY Diversity of structure and function of response regulator output domains Curr Opin Microbiol 2010 13 150 9 10.1016/j.mib.2010.01.005 20226724 PMC3086695

[B53] Maris AE Walthers D Mattison K Byers N Kenney LJ The response regulator OmpR oligomerizes via beta-sheets to form head-to-head dimers J Mol Biol 2005 350 843 56 10.1016/j.jmb.2005.05.057 15979641

[B54] Toro-Roman A Mack TR Stock AM Structural analysis and solution studies of the activated regulatory domain of the response regulator ArcA: a symmetric dimer mediated by the alpha4-beta5-alpha5 face J Mol Biol 2005 349 11 26 10.1016/j.jmb.2005.03.059 15876365 PMC3690759

[B55] Toro-Roman A Wu T Stock AM A common dimerization interface in bacterial response regulators KdpE and TorR Protein Sci 2005 14 3077 88 10.1110/ps.051722805 16322582 PMC2253231

[B56] Gupta S Pathak A Sinha A Sarkar D Mycobacterium tuberculosis PhoP recognizes two adjacent direct-repeat sequences to form head-to-head dimers J Bacteriol 2009 191 7466 76 10.1128/JB.00669-09 19820095 PMC2786591

[B57] Igarashi K Hanamura A Makino K Aiba H Mizuno T Nakata A Ishihama A Functional map of the alpha subunit of Escherichia coli RNA polymerase: two modes of transcription activation by positive factors 1991 88 8958 62 10.1073/pnas.88.20.8958 PMC52630 1833768

[B58] Kondo H Nakagawa A Nishihira J Nishimura Y Mizuno T Tanaka I Escherichia coli positive regulator OmpR has a large loop structure at the putative RNA polymerase interaction site Nat Struct Biol 1997 4 28 31 10.1038/nsb0197-28 8989318

[B59] Martinez-Hackert E Stock AM The DNA-binding domain of OmpR: crystal structures of a winged helix transcription factor Structure 1997 5 109 24 10.1016/S0969-2126(97)00170-6 9016718

[B60] Pratt LA Silhavy TJ OmpR mutants specifically defective for transcriptional activation J Mol Biol 1994 243 579 94 10.1016/0022-2836(94)90033-7 7966283

[B61] Russo FD Slauch JM Silhavy TJ Mutations that affect separate functions of OmpR the phosphorylated regulator of porin transcription in Escherichia coli J Mol Biol 1993 231 261 73 10.1006/jmbi.1993.1281 8389883

[B62] Sharif TR Igo MM Mutations in the alpha subunit of RNA polymerase that affect the regulation of porin gene transcription in Escherichia coli K-12 J Bacteriol 1993 175 5460 8 8396118 10.1128/jb.175.17.5460-5468.1993 PMC206602

[B63] Slauch JM Russo FD Silhavy TJ Suppressor mutations in rpoA suggest that OmpR controls transcription by direct interaction with the alpha subunit of RNA polymerase J Bacteriol 1991 173 7501 10 1657891 10.1128/jb.173.23.7501-7510.1991 PMC212516

[B64] Makino K Amemura M Kawamoto T Kimura S Shinagawa H Nakata A Suzuki M DNA binding of PhoB and its interaction with RNA polymerase J Mol Biol 1996 259 15 26 10.1006/jmbi.1996.0298 8648643

[B65] Kim SK Makino K Amemura M Nakata A Shinagawa H Mutational analysis of the role of the first helix of region 4.2 of the sigma 70 subunit of Escherichia coli RNA polymerase in transcriptional activation by activator protein PhoB Mol Gen Genet 1995 248 1 8 10.1007/BF02456607 7651320

[B66] Makino K Amemura M Kim SK Nakata A Shinagawa H Role of the sigma 70 subunit of RNA polymerase in transcriptional activation by activator protein PhoB in Escherichia coli Genes Dev 1993 7 149 60 10.1101/gad.7.1.149 8422984

[B67] Mattison K Oropeza R Kenney LJ The linker region plays an important role in the interdomain communication of the response regulator OmpR J Biol Chem 2002 277 32714 21 10.1074/jbc.M204122200 12077136

[B68] Walthers D Tran VK Kenney LJ Interdomain linkers of homologous response regulators determine their mechanism of action J Bacteriol 2003 185 317 24 10.1128/JB.185.1.317-324.2003 12486069 PMC141822

[B69] Pathak A Goyal R Sinha A Sarkar D Domain structure of virulence-associated response regulator PhoP of Mycobacterium tuberculosis: role of the linker region in regulator-promoter interaction(s) J Biol Chem 2010 285 34309 18 10.1074/jbc.M110.135822 20814030 PMC2966044

[B70] Rhee JE Sheng W Morgan LK Nolet R Liao X Kenney LJ Amino acids important for DNA recognition by the response regulator OmpR J Biol Chem 2008 283 8664 77 10.1074/jbc.M705550200 18195018 PMC2417188

[B71] Head CG Tardy A Kenney LJ Relative binding affinities of OmpR and OmpR-phosphate at the ompF and ompC regulatory sites J Mol Biol 1998 281 857 70 10.1006/jmbi.1998.1985 9719640

