# Supplementary material for: Interactions of the CpxA sensor kinase and cognate CpxR response regulator from Yersinia pseudotuberculosis
Source: BMC Res Notes. 2012 Sep 27;5:536. doi: 10.1186/1756-0500-5-536 (PMC3517363; doi:10.1186/1756-0500-5-536)
Supplement: Additional file 1 — Table S1. Bacteria strains and plasmids used in this study. [file 1756-0500-5-536-S1.doc]

**Contents**

**Additional file 1:**

Supplementary Table S1. bacterial strains and plasmids used in this study

**References:**

**Supplementary** Table S1 Bacteria strains and plasmids used in this study

| **Strains or plasmids** | **Genotypes or descriptions** | **Sources or references** |
| --- | --- | --- |
| *E. coli* | | |
| DH5 | F^-^*, rec*A1, *end*A1, *hsd*R17, *sup*E44, *thi-1, gyr*A96*, rel*A1 | Victoria Shingler |
| BTH101 | F^-^*,cya-99, ara*D139*, gal*E15*, gal*K16*, rps*L1(Sp^R^)*, hsd*R2*, mcr*A1*, mcr*B1 | Euromedex |
| JM109 | *end*A1, *rec*A1, *gyr*A96, *thi*, *hsd*R17 (r_k_–, m_k_+), *rel*A1, *sup*E44, Δ(*lac-pro*AB), [F’, *tra*D36, *pro*AB, *laq*I^q^ZΔM15] | Promega |
| *Y. pseudotuberculosis* | | |
| YPIII/pIB102 | *yadA*::Tn*5,* Km^R^ (parental) | [1] |
| Plasmids | | |
| pTZ57T/R | T/A cloning vector | Fermentas |
| pKT25-Zip | Derivative of pKT25, having an leucine zipper motif of GCN4 fused in frame to the C-terminal end of CyaA_T25_, Km^R^ | Euromedex |
| pUT18C-Zip | Derivative of pUT18, having an leucine zipper motif of GCN4 fused in frame to the C-terminal end of CyaA_T18_, Ap^R^ | Euromedex |
| pKT25 | Cloning vector for creating in-frame fusions at the C-terminal end of CyaA_T25_, Km^R^ | Euromedex |
| pKNT25 | Cloning vector for creating in-frame fusions at the N-terminal end of CyaA_T25_, Km^R^ | Euromedex |
| pUT18 | Cloning vector for creating in-frame fusions at the N-terminal end of CyaA_T18_, Ap^R^ | Euromedex |
| pUT18C | Cloning vector for creating in-frame fusions at the C-terminal end of CyaA_T18_, Ap^R^ | Euromedex |
| pJT010 | pKNT25 with full length *cpxA* coding residues 1 to 458 (CpxA_1-458_-CyaA_T25_), Km^R^ | This study |
| pJT009 | pUT18 with full length *cpxA* coding residues 1 to 458 (CpxA_1-458_-CyaA_T18_), Ap^R^ | This study |
| pJK026 | pUT18 with truncated *cpxA* coding residues 1 to 156 (CpxA_1-156_-CyaA_T18_), Ap^R^ | This study |
| pJK016 | pUT18 with truncated *cpxA* coding residues 1 to 240 (CpxA_1-240_-CyaA_T18_), Ap^R^ | This study |
| pJK017 | pUT18 with truncated *cpxA* coding residues 1 to 310 (CpxA_1-310_-CyaA_T18_), Ap^R^ | This study |
| pJK034 | pUT18 with truncated *cpxA* coding residues 157 to 310 (CpxA_157-310_-CyaA_T18_), Ap^R^ | This study |
| pJT003 | pUT18 with truncated *cpxA* coding residues 187 to 458 (CpxA_187-458_-CyaA_T18_), Ap^R^ | This study |
| pJK028 | pUT18 with truncated *cpxA* coding residues 311 to 458 (CpxA_311-458_-CyaA_T18_), Ap^R^ | This study |
| pJT002 | pKT25 with full length *cpxR* coding residues 1 to 232 (CyaA_T25_-CpxR_1-232_), Km^R^ | This study |
| pJK004 | pKT25 with truncated *cpxR* coding residues 1 to 116 residues (CyaA_T25_-CpxR_1-116_), Km^R^ | This study |
| pJK005 | pKT25 with truncated *cpxR* coding residues 1 to 132 residues (CyaA_T25_-CpxR_1-132_), Km^R^ | This study |
| pJK031 | pKT25 with truncated *cpxR* coding residues 132 to 232 residues (CyaA_T25_-CpxR_132-232_), Km^R^ | This study |
| pJK006 | pKT25 with truncated *cpxR* coding residues 117 to 232 residues (CyaA_T25_-CpxR_117-232_), Km^R^ | This study |
| pJK053 | pKT25 with full length *cpxR* coding for D51A (CyaA_T25_-CpxR_D51A_), Km^R^ | This study |
| pJK043 | pKT25 with full length *cpxR* coding for D51E (CyaA_T25_-CpxR_D51E_), Km^R^ | This study |
| pJK048 | pKT25 with near full length *cpxR* coding for Δ11-24 (CyaA_T25_-CpxR_Δ11-24_), Km^R^ | This study |
| pJK038 | pKT25 with near full length *cpxR* coding for Δ117-132 (CyaA_T25_-CpxR_Δ117-132_), Km^R^ | This study |
| pJK054 | pKT25 with near full length *cpxR* coding for Δ188-209 (CyaA_T25_-CpxR_Δ188-209_), Km^R^ | This study |
| pJK001 | pKNT25 with truncated *cpxR* coding residues 1 to 116 (CpxR_1-116_-CyaA_T25_), Km^R^ | This study |
| pJT004 | pUT18 with full length *cpxR* coding residues 1 to 232 (CpxR_1-232_-CyaA_T18_), Ap^R^ | This study |
| pJK007 | pUT18 with truncated *cpxR* coding residues 1 to 116 (CpxR_1-116_-CyaA_T18_), Ap^R^ | This study |
| pJK008 | pUT18 with truncated *cpxR* coding residues 1 to 132 (CpxR_1-132_-CyaA_T18_), Ap^R^ | This study |
| pJK030 | pUT18 with truncated *cpxR* coding residues 134 to 232 (CpxR_134-232_-CyaA_T18_), Ap^R^ | This study |
| pJK009 | pUT18 with truncated *cpxR* coding residues 117 to 232 (CpxR_117-232_-CyaA_T18_), Ap^R^ | This study |
| pJK002 | pKNT25 with truncated *cpxR* coding residues 1 to 132 (CpxR_1-132_-CyaA_T25_), Km^R^ | This study |
| pJK029 | pKNT25 with truncated *cpxR* coding residues 134 to 232 (CpxR_134-232_-CyaA_T25_), Km^R^ | This study |
| pJK003 | pKNT25 with truncated *cpxR* coding residues 117 to 232 (CpxR_117-232_-CyaA_T25_), Km^R^ | This study |
| pKWY2428 | homodimer detection vector containing the N-terminal λcI repressor residues 1 to 131, Cm^R^ | [2] |
| pKWY-YycF(120C) | pKWY2428 with truncated *yycF* encoding C-terminal residues 120 to 235 (λcI_1-131_-YycF_120-235_), Cm^R^ | [2] |
| pJV005 | pKWY2428 with full length *cpxR* encoding 2 to 232 residues (λcI_1-131_-CpxR_2-232_), Cm^R^ | This study |
| pJV019 | pKWY2428 with full length *cpxR* encoding D51A (λcI_1-131_-CpxR_D51A_), Cm^R^ | This study |
| pJV006 | pKWY2428 with full length *cpxR* encoding D51E (λcI_1-131_-CpxR_D51E_), Cm^R^ | This study |
| pJV008 | pKWY2428 with near full length *cpxR* coding for Δ11-24 (λcI_1-131_-CpxR_Δ11-24_), Cm^R^ | This study |
| pJV007 | pKWY2428 with near full length *cpxR* coding for Δ117-132 (λcI_1-131_-CpxR_Δ117-132_), Cm^R^ | This study |
| pJV009 | pKWY2428 with near full length *cpxR* coding for Δ188-209 (λcI_1-131_-CpxR_Δ188-209_), Cm^R^ | This study |
| pJV015 | pKWY2428 with truncated *cpxR* encoding N-terminal residues 2 to 116 (λcI_1-131_-CpxR_2-116_), Cm^R^ | This study |
| pJV016 | pKWY2428 with truncated *cpxR* encoding N-terminal residues 2 to 132 (λcI_1-131_-CpxR_2-132_), Cm^R^ | This study |
| pJV003 | pKWY2428 with truncated *cpxR* encoding C-terminal residues 133 to 232 (λcI_1-131_-CpxR_133-232_), Cm^R^ | This study |
| pJV004 | pKWY2428 with truncated *cpxR* encoding C-terminal residues 117 to 232 (λcI_1-131_-CpxR_117-232_), Cm^R^ | This study |
| pJV010 | pKWY2428 with truncated *cpxR* coding for C-terminal residues 117 to 232 and Δ188-209 (λcI_1-131_-CpxR_117-232, Δ188-209_), Cm^R^ | This study |

**References**

1. I Bölin, H Wolf-Watz: **Molecular cloning of the temperature-inducible outer membrane protein 1 of *Yersinia pseudotuberculosis***. *Infect Immun* 1984, **43**:72-8.

2. T Watanabe, Y Hashimoto, Y Umemoto, D Tatebe, E Furuta, T Fukamizo, K Yamamoto, R Utsumi: **Molecular characterization of the essential response regulator protein YycF in *Bacillus subtilis***. *J Mol Microbiol Biotechnol* 2003, **6**:155-63.
