# Supplementary material for: Interactions of the CpxA sensor kinase and cognate CpxR response regulator from Yersinia pseudotuberculosis
Source: BMC Res Notes. 2012 Sep 27;5:536. doi: 10.1186/1756-0500-5-536 (PMC3517363; doi:10.1186/1756-0500-5-536)
Supplement: Additional file 2 — Table S2. Oligonucleotides used in this study. [file 1756-0500-5-536-S2.doc]

**Contents**

**Additional file 2:**

Supplementary Table S2. Oligonucleotides used in this study

**Supplementary Table S2 Oligonucleotides used in this** study

| **Purpose/vector** | **Oligonucleotide name and sequence** |
| --- | --- |
| BACTH assay | |
| pJT010 | pCpxA-full-XbaI-for, 5´-ACG*TCTAGA*CATGATAAACAGTTTAACGACGCG-3´ and pCpxA-full-EcoRI-rev, 5´-ACG*GAATTC*GAAGATTTAAGCGGATGCAGCGG-3´ |
| pJT009 | pCpxA-full-XbaI-for and pCpxA-full-EcoRI-rev |
| pJK026 | pCpxA-full-XbaI-for and pCpxA(1-156)-full-EcoRI-rev, 5´-ACG*GAATTC*GAAAAATCAGATTGCGGACTATTGGC-3´ |
| pJK016 | pCpxA-full-XbaI-for and pCpxA(1-240)-full-EcoRI-rev, 5´-ACG*GAATTC*GATTGTTGAGCGACGACCATTCTGTC-3´ |
| pJK017 | pCpxA-full-XbaI-for and pCpxA(1-310)-full-EcoRI-rev, 5´-ACG*GAATTC*GAAGCTTTAATGGGTTCGCGGTG-3´ |
| pJK034 | pCpxA(157-310)-full-XbaI-for, 5´-ACG*TCTAGA*CATGATTAATCTGATGTTTGACCGC-3´ and pCpxA(1-310)-full-EcoRI-rev |
| pJT003 | pCpxA-C-terminal-XbaI-for, 5´-ACG*TCTAGA*CATGGCGAAACCTGCCCGCAAGCTG-3´ and pCpxA-full-EcoRI-rev |
| pJK028 | pCpxA(310-458)-full-XbaI-for, 5´-ACG*TCTAGA*CATGGCTGATGAGCTTTGGTCCGATGTG-3´ and pCpxA-full-EcoRI-rev |
| pJT002 | pCpxR-XbaI-for1, 5´-ACG*TCTAGA*CCATAAAATCCTATTAGTTG-3´ and pCpxR-full-Eco(stop)-rev, 5´-ACG*GAATTC*TCATGTTTCTGATACCATCAAG-3´ |
| pJK004 | pCpxR-XbaI-for1 and pCpxR-N-terminal-Eco(stop)-rev, 5´-ACG*GAATTC*TCAGCGTAAAATCGCGCGGAT-3´ |
| pJK005 | pCpxR-XbaI-for1 and pCpxR-N-terminal+Linker-Eco(stop)-rev, 5´-ACG*GAATTC*TCAAGGCGCGCCTTGCTCGGCATT-3´ |
| pJK031 | pCpxR-C-terminal-XbaI-for1, 5´-ACG*TCTAGA*CACACTGGAAGTTGATTGCCTG-3´ and pCpxR-full-Eco(stop)-rev |
| pJK006 | pCpxR-C-terminal+Linker**-**XbaI-for, 5´-ACG*TCTAGA*CCGCTCCAATTGGAGTGAACAG-3´ and pCpxR-full-Eco(stop)-rev |
| pJK053 | pCpxR-XbaI-for1 and pCpxR-full-Eco(stop)-rev |
| pJK043 | pCpxR-XbaI-for1 and pCpxR-full-Eco(stop)-rev |
| pJK048 | pCpxR-XbaI-for1 and pCpxR-full-Eco(stop)-rev |
| pJK038 | pCpxR-XbaI-for1 and pCpxR-full-Eco(stop)-rev |
| pJK054 | pCpxR-XbaI-for1 and pCpxR-full-Eco(stop)-rev |
| pJK001 | pCpxR-XbaI-for, 5´-ACG*TCTAGA*CATGCATAAAATCCTATTAGTTG-3´ and pCpxR-N-terminal-EcoRI-rev, 5´-ACG*GAATTC*GAGCGTAAAATCGCGCGGATACGA-3´ |
| pJT004 | pCpxR-XbaI-for and pCpxR-full-EcoRI-rev, 5´-ACG*GAATTC*GATGTTTCTGATACCATCAAGTAG-3´ |
| pJK007 | pCpxR-XbaI-for and pCpxR-N-terminal-EcoRI-rev |
| pJK008 | pCpxR-XbaI-for and pCpxR-N-terminal-linker-EcoRI-rev, 5´-ACG*GAATTC*GAAGGCGCGCCTTGCTCGGCATT-3´ |
| pJK030 | pCpxR-C-terminal-XbaI-for, 5´-ACG*TCTAGA*CATGACACTGGAAGTTGATTGCCTG-3´ and pCpxR-full-EcoRI-rev |
| pJK009 | pCpxR-C-terminal-Linker-XbaI-for, 5´-ACG*TCTAGA*CATGCGCTCCAATTGGAGTGAACAG-3´ and pCpxR-full-EcoRI-rev |
| pJK002 | pCpxR-XbaI-for and pCpxR-N-terminal-linker-EcoRI-rev |
| pJK029 | pCpxR-C-terminal-XbaI-for and pCpxR-full-EcoRI-rev |
| pJK003 | pCpxR-C-terminal-Linker-XbaI-for, 5´-ACG*TCTAGA*CATGCGCTCCAATTGGAGTGAACAG-3´ and pCpxR-full-EcoRI-rev |
| λcI homodimerization assay | |
| pJV005 | pCpxR-full(λ-cl)-for, 5´-GGA*AGATCT*TCCACATAAAATCCTATTAGTTGATGATGA-3´ and pCpxR-full(λ-cl)-rev, 5´-CGG*GGTACC*CCGTCATGTTTCTGATACCATCAA-3´ |
| pJV019 | pCpxR-full(λ-cl)-for and pCpxR-full(λ-cl)-rev |
| pJV006 | pCpxR-full(λ-cl)-for and pCpxR-full(λ-cl)-rev |
| pJV008 | pCpxR-full(λ-cl)-for and pCpxR-full(λ-cl)-rev |
| pJV007 | pCpxR-full(λ-cl)-for and pCpxR-full(λ-cl)-rev |
| pJV009 | pCpxR-full(λ-cl)-for and pCpxR-full(λ-cl)-rev |
| pJV015 | pCpxR-full(λ-cl)-for and pCpxR-N-ter-rev, 5´-CGG*GGTACC*CCGATTGCGTAAAATCGCGCGGAT-3´ |
| pJV016 | pCpxR-full(λ-cl)-for and pCpxR-N-ter+L-rev, 5´-CGG*GGTACC*CCGATTAGGCGCGCCTTGCTC-3´ |
| pJV003 | pCpxR-C-ter(λ-cl)-for, 5´- GGA*AGATCT*TCCAACACTGGAAGTTGATTGCCT -3´ and pCpxR-full(λ-cl)-rev |
| pJV004 | pCpxR-C-ter+L(λ-cl)-for, 5´- GGA*AGATCT*TCCACGCTCCAATTGGAGTGA -3´ and pCpxR-full(λ-cl)-rev |
| pJV010 | pCpxR-C-ter+L(λ-cl)-for and pCpxR-full(λ-cl)-rev |
